# Insufficient Discriminatory Power of Matrix-Assisted Laser Desorption Ionization Time-of-Flight Mass Spectrometry Dendrograms to Determine the Clonality of Multi-Drug-Resistant *Acinetobacter baumannii* Isolates from an Intensive Care Unit

**DOI:** 10.1155/2015/535027

**Published:** 2015-05-25

**Authors:** John Hoon Rim, Yangsoon Lee, Sung Kuk Hong, Yongjung Park, MyungSook Kim, Roshan D'Souza, Eun Suk Park, Dongeun Yong, Kyungwon Lee

**Affiliations:** ^1^Department of Laboratory Medicine, Severance Hospital, Research Institute of Bacterial Resistance, Yonsei University College of Medicine, Seoul, Republic of Korea; ^2^Department of Laboratory Medicine, Hanyang University College of Medicine, Hanyang University Seoul Hospital, Seoul, Republic of Korea; ^3^Department of Infection Control, Severance Hospital, Seoul, Republic of Korea

## Abstract

While pulsed-field gel electrophoresis (PFGE) is recognized as the gold standard method for clonality analysis, MALDI-TOF MS has recently been spotlighted as an alternative tool for species identification. Herein, we compared the dendrograms of multi-drug-resistant (MDR) *Acinetobacter baumannii* isolates by using MALDI-TOF MS with those by using PFGE. We used direct colony and protein extraction methods for MALDI-TOF MS dendrograms. The isolates with identical PFGE patterns were grouped into different branches in MALDI-TOF MS dendrograms. Among the isolates that were classified as very close isolates in MALDI-TOF MS dendrogram, PFGE band patterns visually showed complete differences. We numeralized similarity among isolates by measuring distance levels. The Spearman rank correlation coefficient values were 0.449 and 0.297 between MALDI-TOF MS dendrogram using direct colony and protein extraction method versus PFGE, respectively. This study is the first paper focusing solely on the dendrogram function of MALDI-TOF MS compared with PFGE. Although MALDI-TOF MS is a promising tool to identify species in a rapid manner, our results showed that MALDI-TOF MS dendrograms could not substitute PFGE for MDR *Acinetobacter baumannii* clonality analysis.

## 1. Introduction

Over the years, various typing methods for the detection of bacterial outbreaks have changed from phenotypic to genomic approaches [1]. Among the various genomic typing methods, pulsed-field gel electrophoresis (PFGE) is recognized as the gold standard [2, 3].

Matrix-assisted laser desorption ionization time-of-flight mass spectrometry (MALDI-TOF MS) has recently been spotlighted as a powerful tool for the identification of a broad spectrum of bacterial species and has now become the major identification tool in clinical microbiology laboratories worldwide [4]. In addition, dendrograms based on main spectrum projection (MSP) using MALDI-TOF MS are provided without any cost or additional manual procedures. However, this function was evaluated only in a limited number of bacterial species, such as* Klebsiella pneumonia* [5], vancomycin-resistant enterococci [6],* Escherichia coli* [7], and* Listeria *species [8].

Multi-drug-resistant (MDR)* Acinetobacter baumannii* (*A. baumannii*) outbreaks have been recognized as an increasing threat in hospitals. Numerous nosocomial outbreaks of* A. baumannii*, especially in intensive care units (ICUs), have been reported [9, 10]. The application of MALDI-TOF MS for the rapid detection of carbapenem resistance in a large series of* A. baumannii* clinical isolates has been evaluated [11, 12]; however, the application of* A. baumannii* MALDI-TOF MS dendrograms has never been reported.

Herein, we evaluated whether the MALDI-TOF MS dendrogram is a candidate method to substitute for the current standard PFGE by examining MDR* A. baumannii* clonality based on isolates obtained over a short-term period from an ICU.

## 2. Materials and Methods

### 2.1. Clinical Specimen Collection, Culture, and Species Identification Using MALDI-TOF MS

Thirty MDR* A. baumannii* isolates from respiratory tract specimens (e.g., sputum or endotracheal aspirate) were obtained from 29 patients in an ICU from June 3 to July 14, 2013. In addition, two isolates from ICU environmental samples were included for a total of 32 specimens.

Culture samples were inoculated onto sheep blood and MacConkey agar plates (Asan Pharmaceutical, Seoul, Korea) and then cultured overnight at 37°C in a 5% CO_2_ incubator. Isolates were identified to species using the VITEK 2 GN card and the VITEK 2 system (bioMérieux, Marcy-l'Étoile, France). Antimicrobial susceptibility was determined using AST N212 cards (bioMérieux).

There are several different sample preparation methods for MALDI-TOF MS [13]. Since different sample preparation methods can directly affect results, we implemented the two most commonly used principles, the direct colony and protein extraction methods. We followed the manufacturer's standardized protocols provided by Bruker Daltonics. Bacterial species were confirmed five times on five different days using the direct colony method with MALDI-TOF MS (Bruker Daltonics, Bremen, Germany). In addition, bacterial protein was extracted according to the manufacturer's recommendation. Species identification using the protein extraction method was also repeated five times on five different days to confirm identification score reproducibility.

Since the fundamental principle of the MALDI-TOF MS dendrogram is based on profiling peaks, precision or reproducibility for isolate identification score is very important. For species identification using MALDI-TOF MS, the method included the *m*/*z* from 3,000 to 15,000 Da. For every spectrum, a maximum of 100 peaks were taken into account and compared with spectra in the database. A score enabled the accurate identification and discrimination of the tested species with a score ≥ 2 validating species identification at the species level.

### 2.2. Dendrogram Analysis by MALDI-TOF MS and PFGE

The MSP profile showing the highest score was selected for each isolate and was included to construct the dendrogram using the statistical toolbox in MATLAB 7.1 integrated in the MALDI Biotyper 2.0 software (Bruker Daltonics). Based on the principle that identification score reflects the agreement of the spectra with the standard* A. baumannii* database entry, the MSP profile showing the highest score could mean that the specific spectra represents the most typical aspects of a certain strain from the database. This selection of the highest score marking spectra was necessary, especially when highly similar strains were studied, because several mass spectral features related to limited reproducibility of the method might eclipse mass spectral differences between the strains. Test strain clonality was determined with cut-off values at a distance of 250 [6].

For PFGE analysis of the 32 isolates,* SmaI*-digested genomic DNA was prepared according to the manufacturer's instructions (Bio-Rad, Hercules, CA, USA). Fragments were separated for 20 h at 6.0 V/cm at 11°C using a CHEF-DR II System (Bio-Rad) with initial and final pulse times of 0.5 s and 30 s, respectively [14]. The pattern was analyzed using the Fingerprinting II software (Bio-Rad). The cut-off value of 75 was applied for grouping as it was used previously [15, 16].

### 2.3. Statistics Used to Compare Dendrogram Analysis by MALDI-TOF MS and PFGE

To compare the two clonality analysis methods, we numeralized the relatedness and similarity among the isolates under the consultation of a biostatistics specialist. Specifically, percent identity and distance levels provided in the PFGE and MALDI-TOF MS dendrograms were arithmetically measured, respectively, instead of arbitrarily setting cut-off values for grouping as seen in previous studies [5, 6]. Then, relatedness between all the possible 32 isolate pairs was measured by numerically quantifying distances at which the specific two isolates were separated. To compensate the absolute values compared in different units, a ratio of the distances was used with the first branching distance being the denominator. Since relatedness could be obtained by subtracting distances between specific two isolates, we calculated all relatedness between every possible combination among the 32 isolates. The Spearman rank correlation coefficient and dot plot were obtained using PASW Statistics software (version 18.0, SPSS Inc., Chicago, IL, USA). All the statistical analyses were confirmed by a medical statistics specialist.

## 3. Results

### 3.1. Isolate Characteristics

All sputum and endotracheal aspirate specimens satisfied the criteria of acceptable sputum quality based on a modified Washington and Murray score system [17]. Characteristics of patients including age, sex, major diagnosis for ICU admission, and underlying diseases are shown in Table 1. Although all 32 isolates were defined as MDR* A. baumannii*, antibiotic susceptibility test profiles were slightly different.

### 3.2. Species Identification by MALDI-TOF MS with Two Different Sample Preparation Methods

With repeated MALDI-TOF MS identification, 15 isolates showed higher scores by the direct colony method than the protein extraction method among replicated MSP of each strain, whereas 17 isolates showed higher identification scores by the protein extraction method than the direct colony method.

### 3.3. Dendrograms by MALDI-TOF MS and PFGE

**Figure 1:**
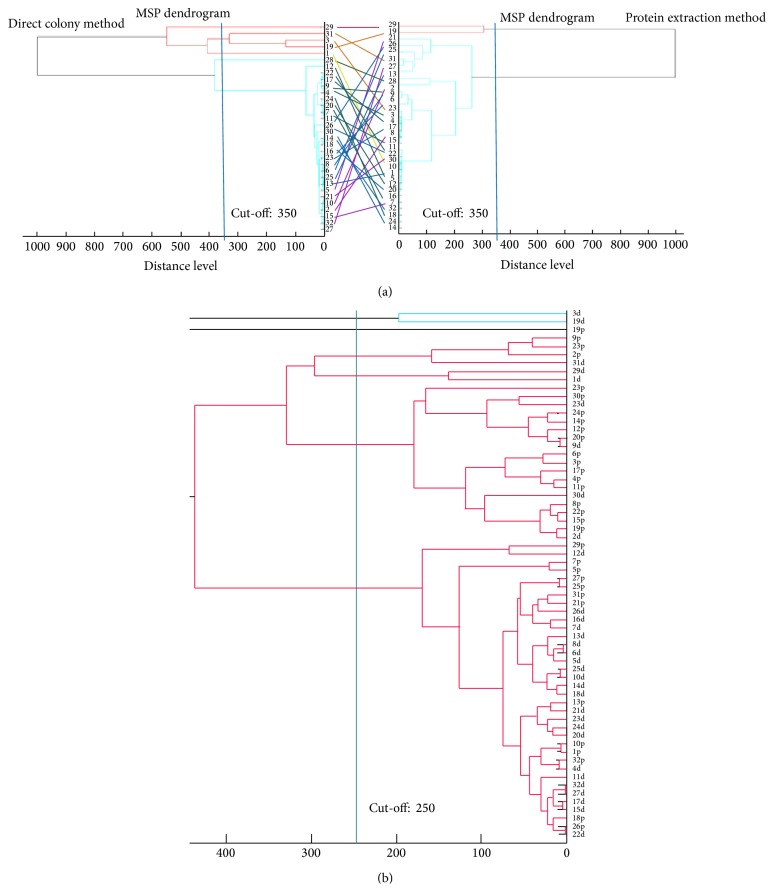
MALDI-TOF MS dendrograms for tested isolates. (a) Comparison between MALDI-TOF MS dendrograms with a cut-off value of 350 using the direct colony method (left) and the protein extraction method (right). (b) MALDI-TOF MS dendrogram of all 64 isolates (32 isolates identified by the two methods) with a cut-off value of 250.

**Figure 2:**
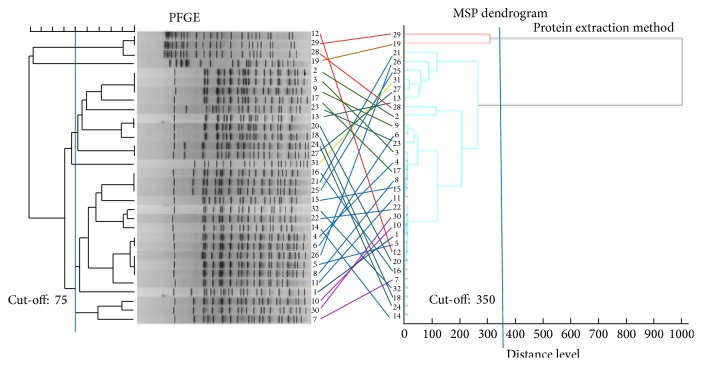
Comparison between PFGE (left) and the MALDI-TOF MS dendrogram using the protein extraction method (right).

Discordance between the two different MALDI-TOF MS dendrograms based on different sample preparation methods (direct colony method versus protein extraction method) was obvious (Figure 1(a)). With the same cut-off value at a distance level of 350, the direct colony method generated five clusters whereas the protein extraction method generated only two clusters. When all 64 MSP identification data with maximum scores based on the two preparation methods in the 32 test strains were put into one dendrogram (Figure 1(b)), only 37.5% of the isolate pairs were classified into the same group with a cut-off value of 250 distance level. PFGE classified the 32 isolates into six groups with a cut-off value of 75 (Figure 2). The isolates that had identical PFGE patterns were grouped into different branches based on the MALDI-TOF MS dendrogram. Among the isolates that were classified as very close isolates using the MALDI-TOFMS dendrogram, PFGE band patterns visually showed complete differences when analyzed using the PFGE interpretation guideline suggested by Tenover et al. [18].

### 3.4. Comparison between MALDI-TOF MS Dendrogram and PFGE

**Figure 3:**
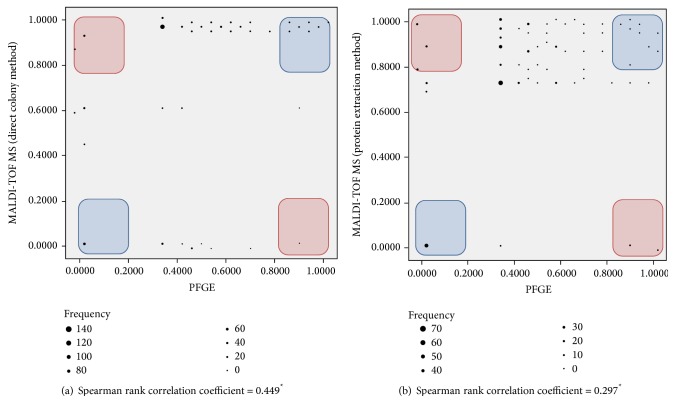
Comparison between PFGE and the MALDI-TOF MS dendrogram using the Spearman rank correlation analysis and dot plots. (a) Comparison between PFGE and the MALDI-TOF MS dendrogram using the direct colony method and (b) comparison between PFGE and the MALDI-TOF MS dendrogram using the protein extraction method.  ^*^Statistically significant (*P* < 0.001).

The Spearman rank correlation coefficient was 0.449 (*P* < 0.001) between PFGE and the MALDI-TOF MS dendrogram using the direct colony method and was 0.297 (*P* < 0.001) between PFGE and the MALDI-TOF MS dendrogram using the protein extraction method, which indicates only slight correlation between them. Measurement of closeness using dot plots between the isolates showed no linear correlation (Figure 3). With 20% or 80% as the cut-off values for acceptable identical or different interisolate distances, respectively, 16.1% showed similar relatedness while 6.1% revealed totally opposite results in comparison between PFGE and the MALDI-TOF MS dendrogram using the direct colony method (Figure 3(a)). With the same cut-off values, 14.3% showed similar relatedness while 4.8% revealed totally opposite results in comparison between PFGE and the MALDI-TOF MS dendrogram using the protein extraction method (Figure 3(b)). These data suggest that correlation between PFGE and MALDI-TOF MS dendrogram results is not strong.

## 4. Discussion

Since MALDI-TOF MS is now routinely utilized in clinical microbiology laboratories in many countries, many studies have been published emphasizing the advantages of MALDI-TOF MS. Berrazeg et al. [5] showed that the MALDI-TOF MS dendrogram, as a promising tool to identify species, could differentiate between MDR* Klebsiella pneumoniae* clinical isolates according to their phenotypic properties and epidemiological distribution. However, among a total of 535 isolates, MALDI-TOF MS dendrogram of only 28 isolates was compared with multilocus sequence typing (MLST). Also, the arbitrarily set cut-off distances of 500, 180, and 100 could be questioned, along with the minimum MALDI-TOF MS identification score criterion of 1.90 that was used in the study.

Wang et al. [19] reported interesting findings that MALDI-TOF MS dendrogram of* Streptococcus pyogenes* species matched well with the determination of M serotypes prevalent in China and was therefore declared suitable for clonality analysis. However, only one specific cluster was interpreted as important without further explanation for the other clusters identified in the study. Hence, they concluded their study with a statement that advances in MALDI-TOF MS technology are needed to improve accuracy for species classification.

Despite these previous studies suggesting that the MALDI-TOF MS dendrogram could serve as a powerful tool in clonality analysis [11, 20], this study is the first paper focusing solely on the dendrogram function of MALDI-TOF MS compared with PFGE, concluding that it has insufficient discriminatory power for MDR* A. baumannii* outbreak strain analysis. Our study is consistent with a recent finding that the discriminatory power of MALDI-TOF MS was found to be insufficient for reliably subdifferentiating* Enterococcus faecium* and* Staphylococcus aureus* isolates to the level of distinct clones or clonal complexes assessed by MLST [21].

An issue with the comparison of proteomic methods to genomic methods is that the presence of genotype does not always correlate linearly with the phenotype. Since current epidemiological tools for strain classification are mainly based on genetic features, MALDI-TOF MS could be outstanding as a proteomic approach for phenotypic analysis.

When we narrow scope of the research to the utility of MALDI-TOF MS for* A. baumannii* species, there are recent studies that indicate the improved discriminatory power of MALDI-TOF MS dendrogram to differentiate* A. baumannii* from* Acinetobacter calcoaceticus* complex strains based on a good identification of* A. baumannii* species [22, 23]. However, our results focus on the clonality analysis among the same species, not the same isolates at the genus level. Spinali et al. [24] showed fairly good correlation between MALDI-TOF MS typing and PFGE for* Streptococcus agalactiae*,* Streptococcus pyogenes*,* Streptococcus pneumonia*, and* Staphylococcus aureus*, but the authors insist that more work is needed to better define interstrain relationships for* A. baumannii*.

We also need to consider the fact that MALDI-TOF MS sample preparation methods, culture medium type, centrifuge speed, and even analysis software programs have not yet been standardized [25]. These issues can result in poor reproducibility for identification scores and even in specific minor peaks, which could change dendrogram grouping. Even though protein extraction methods are recommended for species identification [13], our results suggest that the direct colony method could produce relatively high identification scores compared to the protein extraction method. Interestingly, 62.5% of isolates in the MSP profile using different sample preparation methods did not cluster into one group in the MALDI-TOF MS dendrogram, indicating that MALDI-TOF MS has low reproducibility. There are also some studies that use individually modified procedures other than the direct colony method or the protein extraction method [6, 26]. In addition, types of culture medium were shown to affect MALDI-TOF MS identification results and scores in this study. The choice of blood agar could be an important factor for high score in identification analysis, because colonies grown on MacConkey agar did not result in high identification scores.

Here, we propose new measurements of comparison between clonality analysis methods that could be a remedy for arbitrary cut-off values used in previous dendrogram studies provided by MALDI-TOF MS (Figure 3). The comparison of clonality analysis methods has been based on visual discrimination since grouping cannot be digitized or quantified. However, the simple and highly applicable method used in this study could be applied to MALDI-TOF MS dendrograms as well as PFGE, because branching points can be highlighted. The Spearman rank correlation coefficients of 0.449 and 0.297 (*P* < 0.001) with randomly scattered dot plots indicate that there is no correlation between distances measured from MALDI-TOF MS dendrograms and PFGE.

There are some limitations to this study. First, only 32 isolates in the ICU outbreak were included in this clonality analysis. Taking into consideration that such a short period (42 days) of specimen collection in one ICU was used, the 32 isolates could realistically be a large enough number of isolates to suspect an outbreak and to be used to investigate clonality analysis. Secondly, other clonality analysis methods such as MLST or whole genome sequencing could be compared with MALDI-TOF MS dendrograms. Since there are some reports that show discrepancies between PFGE and MLST [27, 28], this study cannot determine whether MALDI-TOF MS dendrograms will correlate well with MLST. Further studies comparing various clonality analyses are required.

## 5. Conclusions

In summary, although MALDI-TOF MS is a promising tool to identify MDR* A. baumannii* species in a rapid manner to recognize an outbreak, our results show that the MALDI-TOF MS dendrograms could not substitute for PFGE in MDR* A. baumannii* clonality analysis. Therefore, the utilization of MALDI-TOF MS to determine clonality of isolates requires cautious insight.

## Figures and Tables

**Table 1:** Characteristics of the isolates tested and ICU patients.

Isolate number	Type of specimen	Sputum grade^*^	Antibiotics (susceptibility/MIC in *μ*g/mL)				Age	Sex	Major disease condition related to respiratory infection	Underlying major diseases	Clinical outcome
			COL	IMI	CFT	LFX					
1	Sputum, endotracheal aspirate	6	S ≤ 0.5	R ≥ 16	R ≥ 64	R ≥ 8	66	F	Pneumonia	Chronic kidney disease, septic arthritis	Discharged
2	Sputum	6	S ≤ 0.5	R ≥ 16	R ≥ 64	R ≥ 8	62	F	None	End-stage renal disease	Discharged
3	Endotracheal aspirate	6	S ≤ 0.5	R ≥ 16	R ≥ 64	R ≥ 8	67	M	Pneumonia	Rectosigmoid junction cancer	Expired
4	Sputum	6	S ≤ 0.5	S ≤ 0.25	S 8	R ≥ 8	29	M	None	Anti-NMDA receptor encephalitis	Discharged
5	Endotracheal aspirate	6	S ≤ 0.5	R ≥ 16	R ≥ 64	R ≥ 8	67	M	Pneumonia	Lung cancer	Expired
6	Endotracheal aspirate	6	S ≤ 0.5	R ≥ 16	R ≥ 64	R ≥ 8	72	F	Pneumonia	Myasthenia gravis, pseudomembranous colitis	Discharged
7	Endotracheal aspirate	6	R 16	R ≥ 16	R ≥ 64	R ≥ 8	61	M	Pneumonia	Pancreatic cyst	Expired
8	Sputum	5	S ≤ 0.5	R ≥ 16	R ≥ 64	R ≥ 8	49	F	Pneumonia	Acute lymphoblastic leukemia	Expired
9	Sputum	6	S ≤ 0.5	R ≥ 16	R ≥ 64	R ≥ 8	2	F	Pneumonia	Hypoxic ischemic encephalopathy	Discharged
10	Endotracheal aspirate	6	S ≤ 0.5	R ≥ 16	R ≥ 64	R ≥ 8	79	F	Pleural effusion, tracheostomy	Aortic regurgitation, chronic renal failure	Discharged
11	Sputum	6	S ≤ 0.5	R ≥ 16	R ≥ 64	R ≥ 8	75	M	Interstitial lung disease	Cholangiocarcinoma, upper GI bleeding	Expired
12^*^	Sputum	6	S ≤ 0.5	R ≥ 16	R ≥ 64	R ≥ 8	71	M	Pneumonia	Urinary tract infection, osteomyelitis, invasive aspergillosis	Discharged
13	Endotracheal aspirate	6	S ≤ 0.5	R ≥ 16	R ≥ 64	R ≥ 8	49	M	None	Diffuse large B-cell lymphoma, bacterial meningitis	Expired
14	Endotracheal aspirate	6	S ≤ 0.5	R ≥ 16	R ≥ 64	R ≥ 8	71	M	None	Alzheimer's disease, pneumothorax	Discharged
15	Endotracheal aspirate	6	S ≤ 0.5	R ≥ 16	R ≥ 64	R ≥ 8	81	M	Pneumonia	Chronic renal failure	Expired
16	Sputum	6	S ≤ 0.5	R ≥ 16	R ≥ 64	R ≥ 8	74	M	Pneumonia	Pneumothorax, acute renal failure	Expired
17	Sputum	6	S ≤ 0.5	R ≥ 16	R ≥ 64	R ≥ 8	78	M	Pneumonia	Acute pulmonary edema	Expired
18	Sputum	6	S ≤ 0.5	R ≥ 16	R ≥ 64	R ≥ 8	31	M	Pneumonia	Aplastic anemia, intracranial hemorrhage	Expired
19	Endotracheal aspirate	6	S ≤ 0.5	R ≥ 16	I 16	I 4	75	F	Pneumonia, pleural effusion	Wegener's granulomatosis	Expired
20	Sputum	6	S ≤ 0.5	R ≥ 16	I 16	I 4	21	M	Pneumonia	Cerebral palsy, Dravet syndrome	Discharged
21	Endotracheal aspirate	6	S ≤ 0.5	R ≥ 16	R ≥ 64	R ≥ 8	62	M	Pneumonia	Gas gangrene	Expired
22	Sputum	6	S ≤ 0.5	R ≥ 16	R ≥ 64	R ≥ 8	45	F	Pneumonia	Systemic sclerosis, ovarian cancer	Expired
23	Sputum	6	S ≤ 0.5	R ≥ 16	R ≥ 64	R ≥ 8	71	M	Pneumonia	Esophageal cancer	Discharged
24	Sputum	6	S ≤ 0.5	R ≥ 16	R ≥ 64	I 4	70	M	Pneumonia	Esophageal cancer	Expired
25	Sputum	6	S ≤ 0.5	R ≥ 16	R ≥ 64	R ≥ 8	43	M	Pneumonia	Cerebral palsy, AV block	Discharged
26	Endotracheal aspirate	6	S ≤ 0.5	R ≥ 16	R ≥ 64	R ≥ 8	75	M	Mediastinitis	Cerebrovascular attack, peptic ulcer	Expired
27	Endotracheal aspirate	6	S ≤ 0.5	R ≥ 16	R ≥ 64	I 4	69	F	None	Hypoxic brain damage	Discharged
28	Sputum	6	S ≤ 0.5	R ≥ 16	R ≥ 64	R ≥ 8	9	F	Pneumonia, pleural effusion	Encephalitis	Inpatient
29^**^	Sputum	6	S ≤ 0.5	R ≥ 16	R ≥ 64	R ≥ 8	71	M	Pneumonia	Urinary tract infection, osteomyelitis, invasive aspergillosis	Discharged
30	Sputum	4	R ≥ 16	R ≥ 16	R ≥ 64	R ≥ 8	66	M	Pneumonia	Anaplastic astrocytoma, atrial septal defect	Discharged
31^***^	—	—	S ≤ 0.5	R ≥ 16	R ≥ 64	R ≥ 8	—	—	—	—	—
32^***^	—	—	S ≤ 0.5	R ≥ 16	R ≥ 64	R ≥ 8	—	—	—	—	—

^*^Sputum grade is assessed based on a modified Washington and Murray score system.

^**^Isolates numbers 12 and 29 were collected from the same patient at different times during hospitalization.

^***^Environment surveillance specimens (monitor cable line between beds).

MIC, minimal inhibitory concentration; COL, colistin; IPM, imipenem; CTX, cefotaxime; LEV, levofloxacin; S, susceptible; R, resistant; I, intermediate.
